# Anxiety Symptoms and Risk of Dementia Progression in Mild Cognitive Impairment: A Systematic Review and Meta-Analysis

**DOI:** 10.1159/dem/aciag007

**Published:** 2026-09-25

**Authors:** David Fresnais, Brynjar Fure

**Affiliations:** a School of Medical Sciences, Örebro University, Örebro, Sweden; b Department of Internal Medicine, Central Hospital Karlstad, Karlstad, Sweden

**Keywords:** Behavioral disturbance, Anxiety, Mild cognitive impairment, Dementia, Alzheimer’s disease

## Abstract

**Introduction:**

Anxiety is among the earliest and most common neuropsychiatric symptoms in cognitive impairment. Emerging evidence suggests that anxiety may elevate the risk of cognitive decline in mild cognitive impairment (MCI). This systematic review and meta-analysis was performed to quantitatively synthesize evidence from cohort studies on the association between anxiety symptoms and dementia conversion in MCI.

**Methods:**

A systematic literature search was performed in Medline and other relevant databases to find cohort studies examining the association between anxiety symptoms and dementia progression in MCI.

**Results:**

Fifteen cohort studies were included in the systematic review, of which fourteen were included in meta-analyses. Anxiety was significantly associated with an increased risk of all cause dementia, hazard ratio (HR) = 1.25; 95% confidence interval (CI): 1.11–1.41; *p* < 0.001, and Alzheimer’s disease, HR = 1.32; 95% CI: 1.16–1.49; *p* < 0.001, while pooled odds ratios did not show a significant association odds ratio = 1.15; 95% CI: 0.84–1.57; *p* = 0.39.

**Conclusion:**

Anxiety symptoms may be associated with an elevated risk of dementia conversion in MCI. Further large-scale prospective cohort studies are needed to determine the strength and clinical importance of these findings.

## Introduction

Mild cognitive impairment (MCI) is a risk state for progression to dementia and is diagnosed by cognitive impairment ≥1.5 standard deviations (SDs) below age- and education-adjusted norms, in the absence of impaired activities of daily living [1]. Neuropsychiatric symptoms (NPS), including depression, anxiety, apathy, and sleep disturbances, are almost invariably seen over the course of dementia and may precede cognitive symptoms [2]. In the former context, these symptoms are referred to as behavioral and psychological symptoms of dementia [3], whereas their occurrence prior to dementia onset is termed mild behavioral impairment [4]. Identification of NPS in persons evaluated for cognitive impairment is therefore of clinical interest, as these symptoms may have prognostic value and could represent potentially modifiable targets for early intervention.

NPS can be assessed using instruments such as the Neuropsychiatric Inventory Questionnaire (NPI-Q), which captures 12 symptom domains, including depression, anxiety, elation, apathy, irritability, agitation, disinhibition, sleep and appetite disturbances, delusions, hallucinations, and motor disturbances [5]. Anxiety is common among individuals with cognitive impairment [6, 7], yet it has received comparatively less attention than symptoms such as depression or apathy. This may partly be explained by anxiety historically being regarded as a component of depressive symptomatology rather than a separate clinical entity. Although anxiety and depression often coexist [8], anxiety symptoms are now classified separately in both the NPI-Q and other validated instruments, such as the Hospital Anxiety and Depression Scale (HADS) [9].

The relationship between anxiety and Alzheimer’s disease (AD) was summarized in a systematic review published in 2021, which reported that anxiety symptoms are frequently observed early in the course of cognitive impairment and are associated with conversion to dementia [10]. The biological mechanisms underlying this association remain incompletely understood, although anxiety symptoms have been linked to both neurodegenerative [11] and microvascular brain pathology [12]. A systematic review and meta-analysis published in 2018 reported an elevated hazard ratio (HR) for dementia conversion associated with anxiety symptoms in persons with MCI [13]. However, longitudinal studies published since then have shown conflicting results regarding the association between anxiety symptoms and progression to dementia [14, 15]. The present systematic review and meta-analysis was conducted to update the existing literature and quantitatively examine the association between anxiety symptoms and progression to dementia among persons with MCI using data from longitudinal cohort studies.

## Methods

### Search Strategy

A systematic literature search was performed in Medline, EMBASE, Cochrane Library, PsycINFO, CINAHL, and Epistemonikos to find longitudinal cohort studies examining the association between anxiety symptoms and progression to dementia in persons with MCI. The search strategy was built on an existing search strategy from a previous systematic review and meta-analysis by the same authors [16] and was updated to include studies published after the original search. The search was performed in September 2025 and was limited to studies published from 2000 onward and to articles written in English or Scandinavian languages. For the full search strategy, see online supplementary Information 1 and 2 (for all online suppl. material, see https://doi.org/10.1159/dem/aciag007).

### Selection Criteria

This systematic review and meta-analysis was performed in accordance with the Preferred Reporting Items for Systematic Reviews and Meta-Analyses (PRISMA) guidelines [17], see online supplementary information 4 for the complete PRISMA Checklist. After removal of duplicate records, two investigators independently screened titles and abstracts for potential eligibility. Selected articles were subsequently assessed in full text and had to meet the following prespecified PECOS criteria for inclusion: population – persons with MCI; exposure – assessment of anxiety symptoms using validated instruments or criteria; controls – persons with MCI without anxiety symptoms; outcome – progression to dementia; and study design – longitudinal cohort studies. Studies focusing on stroke populations or conditions unrelated to cognitive impairment were excluded, as were studies that did not report a specific association between anxiety symptoms and dementia progression.

### Data Extraction and Analysis

Data were extracted by investigator DF and independently verified by investigator BF. Extracted variables included: (1) HRs for dementia progression among persons with MCI and anxiety symptoms; (2) prevalence and severity of anxiety symptoms and the instruments used for assessment; (3) diagnostic criteria for MCI and dementia; and (4) general study characteristics, including year of publication, country and research setting, study design, participant demographics, and duration of follow-up. Adjusted HRs were used for the primary analysis when available, although the variables included in adjustment models varied between studies. Subgroup analyses were performed for AD as dementia etiology, as well as analyses of standardized mean differences (SMDs) in anxiety symptoms between converters and non-converters. The SMD allows for comparison of mean differences between groups across studies using varying scales and diagnostic criteria.

### Statistics

All statistical analyses were performed using Review Manager version 5.4 [18]. The primary analysis pooled adjusted HRs to estimate the risk of dementia progression associated with anxiety symptoms in persons with MCI. Study weights were assigned automatically by the software. Additional analyses examined mean anxiety symptom scores and differences in the proportion of participants presenting with anxiety symptoms between converters and non-converters. Leaving one out analysis, where the highest weight study was removed, was performed for the primary analysis and a subgroup analysis of AD as dementia etiology. The inverse-variance method was used to pool all meta-analyses, except for the analysis of odds ratios for conversion, for which the Mantel-Haenszel method was used. All analyses employed random-effects models to account for between-study heterogeneity, and two-sided *p* values with 95% confidence intervals (CIs) were used.

### Quality Assessment and Risk of Bias

Study quality and risk of bias were independently assessed by DF and BF using the Critical Appraisal Skills Programme (CASP) checklist for cohort studies [19]. Certainty of evidence for each pooled estimate was evaluated using Grading of Recommendations, Assessment, Development, and Evaluations (GRADE) [20]. A funnel plot was generated for the primary analysis to assess potential publication bias.

### Disagreements between Reviewers

Any disagreements regarding study selection, data extraction, statistical analyses, or quality assessment were resolved through consensus.

## Results

### Literature Search

Following full-text assessment of 93 articles, 15 studies met inclusion criteria and all but one were included in the meta-analyses [6, 7, 14, 15, 21–31], see Figure 1. One of the included studies was identified through a previous systematic review and meta-analysis [26]. One article was excluded because the full text could not be retrieved despite attempts through the hospital library. For more information on reasons for exclusion of studies not meeting eligibility criteria, see online supplementary information 3.

**Fig. 1.**
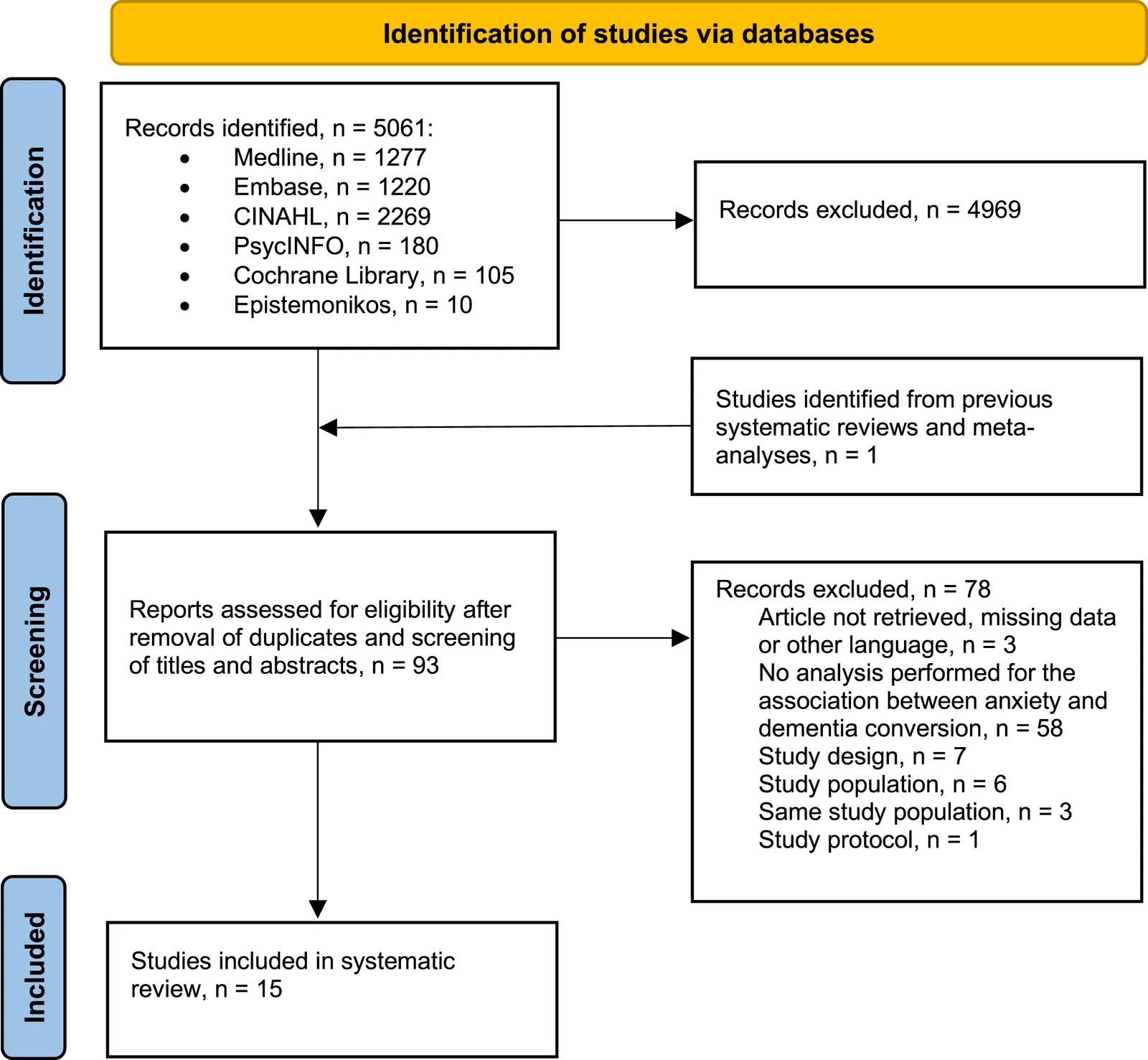
PRISMA flow-chart showing the article inclusion process.

### Study Characteristics

All included studies were longitudinal cohort studies published between 2007 [7] and 2024 [29], see Table 1 for the general characteristics of studies. For information regarding dementia etiology and diagnostic criteria, prevalence of anxiety symptoms, instruments used for assessment of anxiety, rates of progression from MCI to dementia, and reported associations between anxiety symptoms and dementia conversion, see Table 2.

**Table 1.** General characteristics of included studies

Author and year of publication	Country	Setting	Sample type and size	Diagnostic criteria for MCI	Age in years, *N* (SD)	Female, *N* (%)	Years of education, mean (SD)
Brodaty et al. [21] (2014)	Australia	Memory clinics	185 MCI	Petersen criteria	Converted 75.3 (6.7); stable 76.0 (7.0)	85 (45.9)	Education (some tertiary): 91 persons (48.1%)
Cai et al. [14] (2021)	China	Data from ADNI-cohort	699 CN and 701 MCI	ADNI-criteria	Reported according to subgroups	701 (50.0)	Reported according to subgroups
Dietlin et al. [22] (2019)	France	Outpatient memory clinic	96 MCI	RAVLT score <1 SD of mean, MMSE ≥24	78.7 (5.6)	57 (59.4)	8.4(3.9)
Gallagher et al. [23] (2010)	Ireland	Memory clinic	161 MCI	Petersen criteria	Converted 74.7 (5.9); stable 72.9 (7.9)	69 (42.9)	Reported according to cognitive status
Hu et al. [31] (2021)	China	Community-based	441 MCI	Education adjusted MMSE scores, MoCA < 26, intact ADL	68.1 (7.5)	311 (71.8)	55.4% illiterate or primary, 44.6% middle or higher
Mallo et al. [24] (2020)	Spain	Health centers	78 CN and 50 MCI	Petersen criteria	67.3 (8.0)	74 (57.8)	NR
Marquié et al. [15] (2023)	Spain	Memory unit	500 MCI	Petersen criteria	73.3 (7.5)	275 (55)	7.84 (4.8)
Palmer et al. [7] (2007)	Sweden	Population-based	185 CN and 47 MCI	No dementia, intact ADL, objective CI	84 (5.1)	197 (84.9)	39.2% with higher education
Peters et al. [25] (2013)	United States	Population-based study	230 CIND	Clinical diagnosis	81.9 (5.1)	115 (50.0)	13.4 (3.1)
Pink et al. [26] (2015)	USA	Population-based	322 MCI	Revised Mayo Clinic criteria for MCI	82.1 (IQR 77.7, 85.0)	151 (45.5)	12 (IQR 12, 15)
Ramakers et al. [6] (2010)	Netherlands	Memory clinic	263 MCI	Global deterioration scale	66.9 (7.7)	116 (44.0)	Low, middle, high (%): 23/46/31
Rosenberg et al. [27] (2013)	USA	Database of memory clinics	1821 MCI	Clinical diagnosis, Clinical Dementia Rating scale	75.3 (9.3)	920 (50.5)	15.2 (6.2)
Somme et al. [28] (2013)	Spain	Outpatient memory unit	132 MCI	Petersen criteria	69.8 (8.7)	62 (46.9)	10.6 (4.4)
Willmott et al. [29] (2024)	United Kingdom	Mental health records	2250 MCI	ICD-10 criteria	75 (10.8)	1,249 (55.5)	NR
Yang et al. [30] (2020)	China	Population-based	465 MCI	Compound memory score ≤1.5 SD of mean	Converted 75.6 (9.2); stable 80.6 (8.5)	324 (69.7)	Converted 1.8 (3.1); stable 3.5 (3.8)

ADL, activities of daily living; ADNI, Alzheimer’s Disease Neuroimaging Initiative; CI, cognitive impairment; CIND, cognitive impairment no dementia; CN, cognitively normal; ICD-10, International Classification of Diseases, 10th Revision; IQR, interquartile range; MCI, mild cognitive impairment; MMSE, Mini-Mental State Examination; MoCA, Montreal Cognitive Assessment; NR, not reported; RAVLT, Rey Auditory Verbal Learning Test; SD, standard deviation.

**Table 2.** Conversion characteristics and reported associations between anxiety and incident dementia

Author and year of publication	Type of dementia and criteria for diagnosis	Anxiety symptoms at baseline		Follow-up time, mean (SD)	Converters, stable MCI (% with conversion), *N*	Association between anxiety and incident dementia (95% CI)
		tool for detection of anxiety	prevalence in whole sample, *N* (%)			
Brodaty et al. [21] (2014)	All-cause; DSM IV	Chinese version of NPI-Q	10 (5.4)	3 years	52, 133 (28.1)	Mean anxiety scores: converters 1.70 (3.30), stable 1.14 (2.56)
Cai et al. [14] (2021)	AD; NINCDS	NPI-Q	134 (9.6)	3 years	233, 815 (22.2)	Adjusted HR: 1.48 (0.98–2.25), Adjusted OR 1.21 (1.06–1.38)
	ADRDA					
Dietlin et al. [22] (2019)	AD; CDR and clinical	NPI-Q	26 (27.1) “with affective profile”	3.5 years (IQR: 2.9–4)	42, 54 (43.8)	Adjusted HR: 1.56 (0.8–3.03)
Gallagher et al. [23] (2011)	AD; NINCDS	BEHAVE-AD	84 (52)	27 months (18.1)	69, 92 (42.9)	Proportion of anxiety symptoms in conversion group and stable MCI
	ADRDA					
Hu et al. [31] (2021)	NR: MMSE cut-off scores	HAMD	NR	∼6 years	77, 356 (17.8)	Mean differences; converters, non-converters: 0.38 (1.11), 0.35 (0.85)
Mallo et al. [24] (2020)	All-cause; DMS-4-TR	NPI-Q	NR	Up to 6 years	30, 98 (23.4)	Mean anxiety scores: converters 1.03 (1.09), stable 0.52 (0.95)
Marquié et al. [15] (2023)	All-cause; NIA-AA, NINDS-AIREN	NPI-Q	218 (43.6)	2.08 years (1.2)	224, 276 (44.8)	Adjusted HR: 1.04 (0.76–1.4)
Palmer et al. [7] (2007)	All-cause; DSM-III-R	Psychiatrist evaluation	22 (46.8)	3.4 years (0.6)	24, 23 (51.1)	RR: 1.8 (1.2–2.7)
Peters et al. [25] (2013)	All-cause, AD; DSM-III-R	NPI-Q	11 (4.8)	3.3 years	85, 145 (37.8)	Adjusted HR: 0.55 (0.13–2.25)
Pink et al. [26] (2015)	All-cause; DSM-IV	NPI-Q	28 (8.4)	3.0 years (IQR: 2.5–5.3)	117, 215 (35.2)	Adjusted HR: 0.93 (0.54–1.61)
Ramakers et al. [6] (2009)	All cause; NINCDS	HAMD	142 (54)	5.4 years	90, 125 (41.9)	Adjusted OR: 1.7 (0.32–9.01)
	ADRDA and DSM-IV					
Rosenberg et al. [27] (2013)	All-cause, AD; CDR, clinical diagnosis	NPI-Q	353 (19.4)	Median time 1.58	527, 1,294 (28.9)	Adjusted HR: 1.28 (1.12–1.47)
Somme et al. [28] (2013)	AD; NINCDS-ADRDA	Spanish version of NPI-Q	20 (15.2)	3.5 (2.9)	38, 94 (28.8)	Adjusted HR: 2.501 (1.008–6.203)
Willmott et al. [29] (2024)	All-cause; ICD‐10	ICD-10	108 (4.8)	NR	236, 2,014 (10.5)	Adjusted HR: 1.21 (0.66–2.24)
Yang et al. [30] (2020)	AD; DSM-IV-TR, clinical assessment	Chinese version of NPI-Q	55 (11.8)	3	78, 387 (16.8)	Proportion of anxiety symptoms in conversion group and stable MCI

AD, Alzheimer’s disease; ADRDA, Alzheimer’s Disease and Related Disorders Association; BEHAVE-AD, Behavioral Pathology in Alzheimer’s Disease Rating Scale; CDR, Clinical Dementia Rating; CI, confidence interval; DSM-III-R, Diagnostic and Statistical Manual of Mental Disorders, Third Edition, Revised; DSM-IV, Diagnostic and Statistical Manual of Mental Disorders, Fourth Edition; DSM-IV-TR, Diagnostic and Statistical Manual of Mental Disorders, Fourth Edition, Text Revision; HAMD, Hamilton Depression Rating Scale; HR, hazard ratio; ICD-10, International Classification of Diseases, Tenth Revision; IQR, interquartile range; MCI, mild cognitive impairment; MMSE, Mini-Mental State Examination; NIA-AA, National Institute on Aging–Alzheimer’s Association; NINCDS-ADRDA, National Institute of Neurological and Communicative Disorders and Stroke–Alzheimer’s Disease and Related Disorders Association; NINDS-AIREN, National Institute of Neurological Disorders and Stroke–Association Internationale pour la Recherche et l’Enseignement en Neurosciences; NPI-Q, Neuropsychiatric Inventory Questionnaire; NR, not reported; OR, odds ratio; RR, relative risk; SD, standard deviation.

The baseline prevalence of anxiety symptoms ranged from 4.8% [25, 29] to 54% [6]. The NPI-Q was the most frequently used instrument for assessment of NPS. Mean follow-up duration ranged from 1.2 years [27] to 5.4 years [6], and the proportion of participants who progressed to dementia ranged from 10.5% [29] to 51.1% [7]. The Petersen criteria [1] and NINCDS-ADRDA criteria [32] were most used for the diagnosis of MCI and dementia, respectively.

### Meta-Analysis

In the primary meta-analysis pooling HRs from eight studies, anxiety symptoms were associated with an increased risk of progression to all-cause dementia, HR = 1.25; 95% CI: 1.11–1.41; *p* < 0.001, see Figure 2. However, after leaving one out analysis, Rosenberg et al. [27], the analysis of HRs for all cause dementia changed to, HR = 1.22; 95% CI: 0.98–1.51; *p* = 0.08. A subgroup analysis restricted to progression to AD also showed a statistically significant association, HR = 1.32; 95% CI: 1.16–1.49; *p* < 0.001, see Figure 3. This subgroup analysis was still significant after excluding Rosenberg et al. [27] from the analysis, HR = 1.55; 95% CI: 1.12–2.13; *p* = 0.007.

**Fig. 2.**
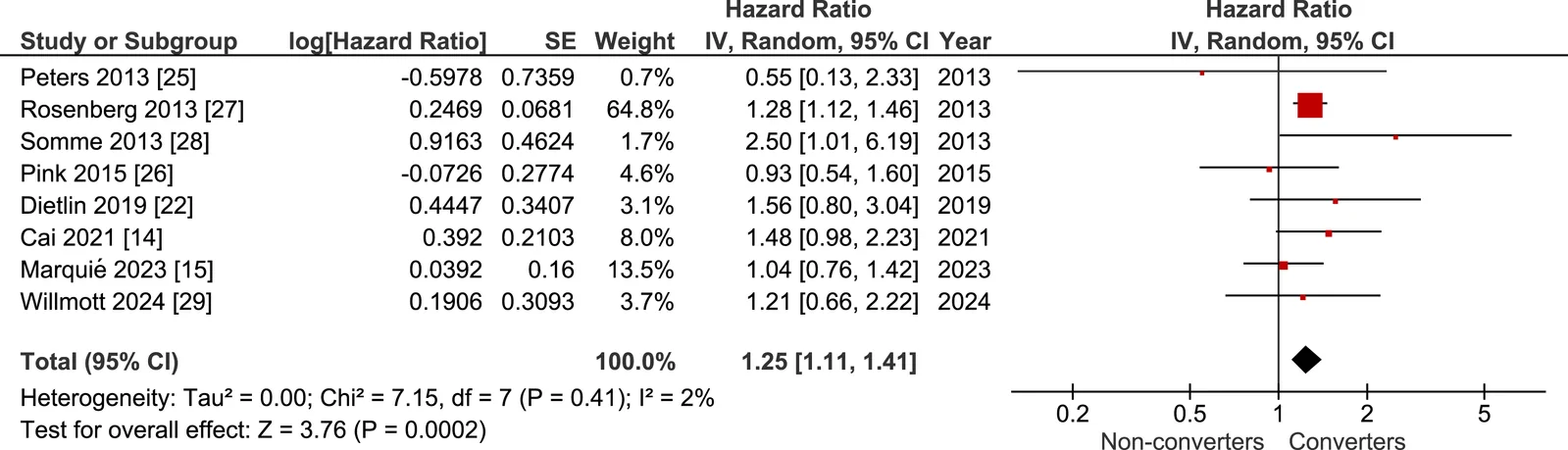
Forest plot showing the pooled HR for the association between anxiety and conversion from MCI to all-cause dementia.

**Fig. 3.**
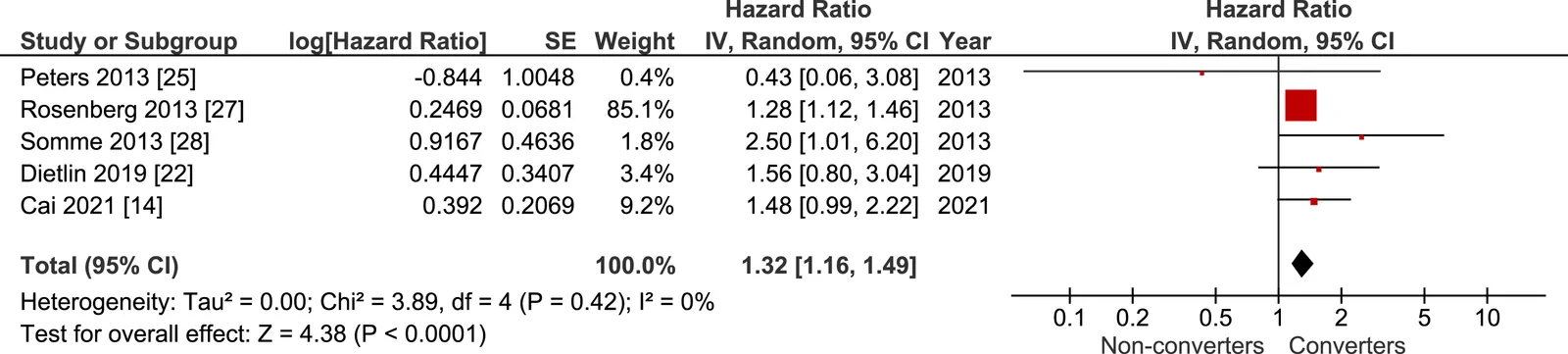
Forest plot showing the pooled HR for the association between anxiety and conversion from MCI to Alzheimer’s disease.

In contrast, comparison of proportions of participants with anxiety symptoms between converters and non-converters did not show a statistically significant association, odds ratio (OR) = 1.15; 95% CI: 0.84 to 1.57; *p* = 0.39, see Figure 4. Analysis of SMD in anxiety symptom scores showed a nonsignificant trend toward higher anxiety symptom severity among converters compared with non-converters, SMD = 0.16; 95% CI: −0.03 to 0.36; *p* = 0.10, see Figure 5.

**Fig. 4.**
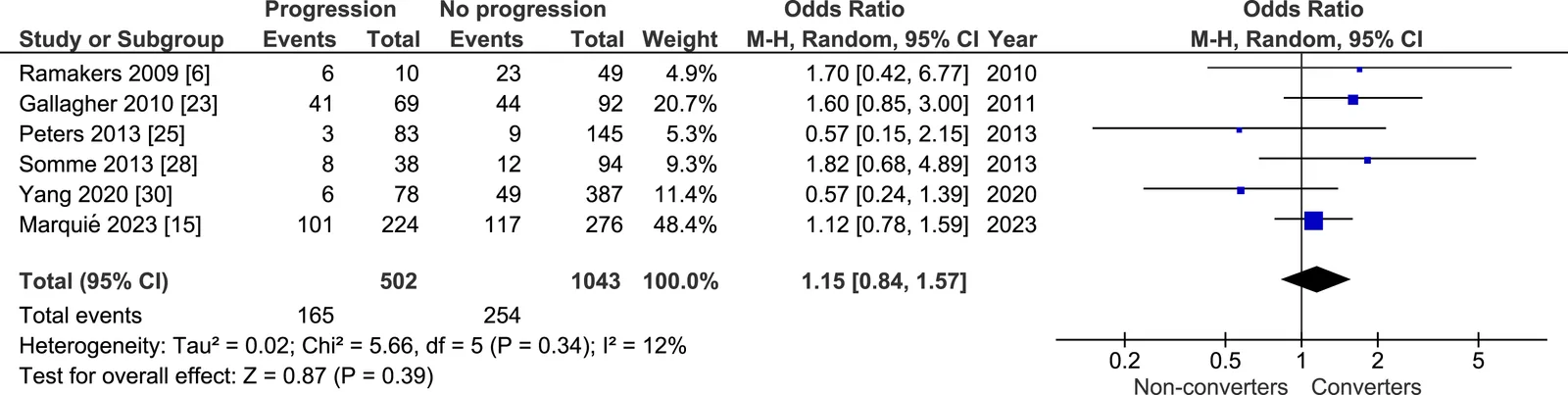
Forest plot showing the pooled odds ratio for the association between anxiety and conversion from MCI to all-cause dementia.

**Fig. 5.**
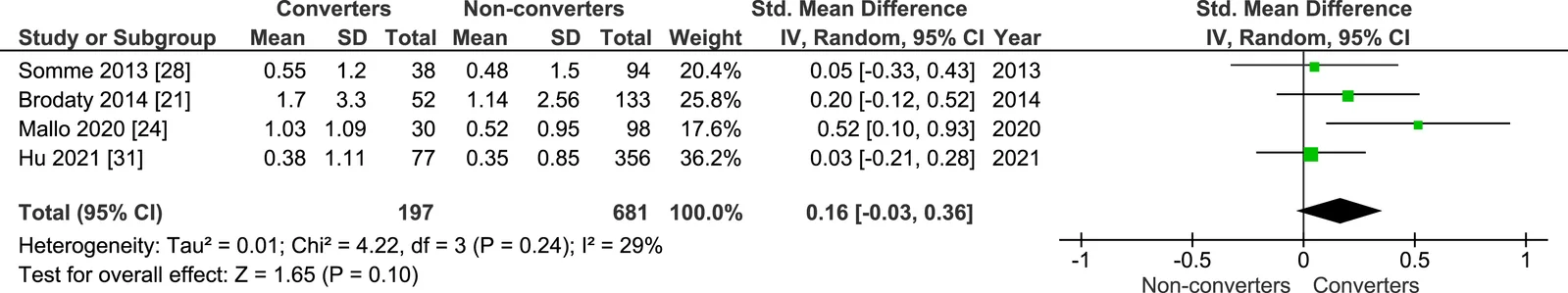
Forest plot showing the pooled standardized mean difference in anxiety symptom scores between persons with MCI who converted to all-cause dementia and those who did not.

### Quality Assessment and Publication Bias

The CASP checklist for cohort studies used for quality assessment and risk of bias for included studies showed a low risk of bias, see Table 3. According to the GRADE framework, the certainty of evidence was rated very low, see Table 4. Visual inspection of the funnel plot for the primary analysis showed no clear asymmetry, but the small number of studies limits interpretation, see Figure 6.

**Table 3.** Quality-assessment of included studies according to critical appraisals skills programme checklist for cohort studies

Author and year of publication	Did the study address a clearly focused issue?	Was the cohort recruited in an acceptable way?	Was the exposure accurately measured to minimize bias?	Was the outcome accurately measured to minimize bias?	Have the authors identified all important confounding factors?	Have they taken account of the confounding factors in the design and/or analysis?	Was the follow up of subjects complete enough?	Was the follow up of subjects long enough?	Do you believe the results?	Can the results be applied to the local population?	Do the results of this study fit with other available evidence?	Overall evaluation of study quality
Brodaty et al. [21] (2014)	+	+	+	+	+	+	−	+	+	+	+	?
Cai et al. [14] (2021)	+	?	+	+	+	+	?	+	+	?	+	?
Dietlin et al. [22] (2019)	+	+	+	+	+	?	+	+	+	+	+	+
Gallagher et al. [23] (2011)	+	?	+	+	?	+	+	+	+	+	+	?
Hu et al. [31] (2021)	+	?	+	?	?	+	?	+	+	?	+	?
Mallo et al. [24] (2020)	+	?	+	+	−	−	?	+	+	+	+	−
Marquié et al. [15] (2023)	+	?	+	+	+	+	+	+	+	+	+	+
Palmer et al. [7] (2007)	+	+	+	+	?	+	+	+	+	+	+	+
Peters et al. [25] (2013)	+	?	+	+	+	+	+	+	+	+	+	+
Pink et al. [26] (2015)	+	+	+	+	+	+	+	+	+	+	+	+
Ramakers et al. [6] (2009)	+	+	+	+	?	+	+	+	+	+	+	+
Rosenberg et al. [27] (2013)	+	?	+	?	+	+	−	+	+	+	+	−
Somme et al. [28] (2013)	+	+	+	+	+	+	+	+	+	+	+	+
Willmott et al. [29] (2024)	+	+	+	+	?	+	+	+	+	?	+	+
Yang et al. [30] (2020)	+	?	+	+	+	+	+	+	+	+	+	+

+, yes or good; −, no or not good; ?, uncertain.

**Table 4.** GRADE evidence table presenting assessments of certainty in the pooled estimates

Certainty assessment							Patients, *n*		Effect		Certainty	Importance
studies, *n*	study design	risk of bias	inconsistency	indirectness	imprecision	other considerations	converters	non-converters	relative (95% CI)	absolute (95% CI)		
*HR for the association between anxiety and conversion to all-cause dementia*												
8	Non-randomized studies	Serious^a^	Not serious	Not serious	Serious^b^	None	​	​	**HR 1.25** (1.11–1.41) [HR for dementia conversion]	​	⨁◯◯◯	IMPORTANT
											Very low^a^^,^^b^	
*HR for the association between anxiety and conversion to AD*												
5	Non-randomized studies	Serious^a^	Not serious	Not serious	Serious^b^	None	​	​	**HR 1.32** (1.16–1.49) [HR for conversion to AD]	​	⨁◯◯◯	IMPORTANT
											Very low^a^^,^^b^	
*Odds ratio for the association between anxiety and conversion to all-cause dementia*												
6	Non-randomized studies	Serious^a^	Serious^c^	Not serious	Serious^b^	None	502 participants	1,043 participants	**OR 1.15** (0.84–1.57) [OR for dementia conversion]	​	⨁◯◯◯	IMPORTANT
											Very low^a^^,^^b^^,^^c^	
*Standardized mean difference in anxiety symptom scores between converters and non-converters to all-cause dementia*												
4	Non-randomized studies	Serious^a^	Not serious	Not serious	Serious^b^	None	197	681	–	SMD **0.16 SD higher** (0.03 lower to 0.36 higher)	⨁◯◯◯	IMPORTANT
											Very low^a^^,^^b^	

CI, confidence interval; HR, hazard ratio; OR, odds ratio; SMD, standardized mean difference.

^a^ Risk of bias.

^b^ Broad CIs.

^c^ Effect estimates in opposite directions.

**Fig. 6.**
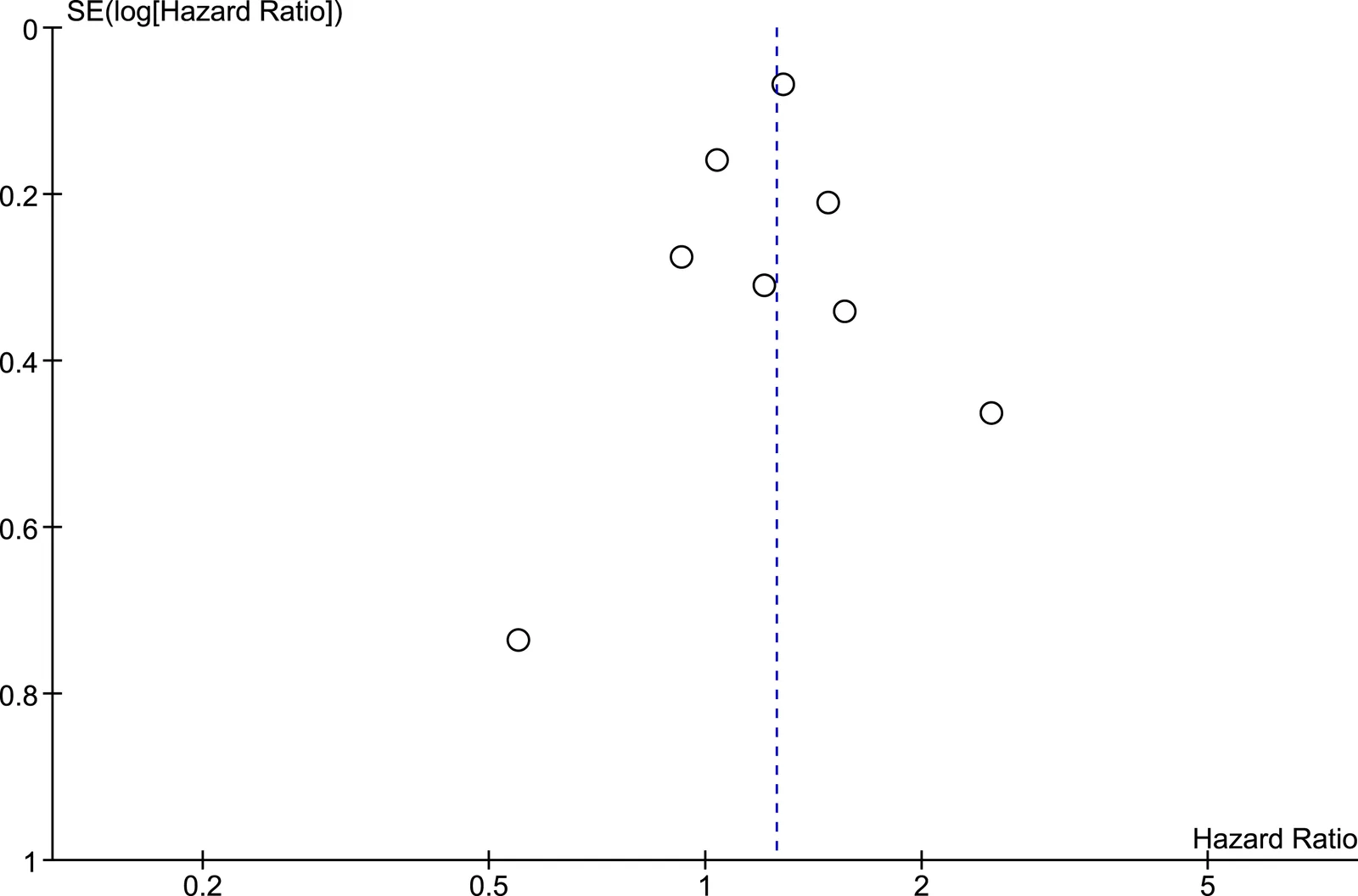
Funnel-plot for assessment of publication bias in the meta-analysis of the pooled HR for the association between anxiety and conversion from MCI to all-cause dementia. The funnel-plot shows no clear asymmetry, although interpretation is limited by the small number of studies.

## Discussion

In this systematic review and meta-analysis of longitudinal cohort studies, anxiety symptoms were associated with an increased risk of progression from MCI to dementia. This association was observed both for all-cause dementia and for AD specifically. In addition, there was a trend toward higher anxiety symptom severity among individuals who converted to dementia compared with non-converters, although this finding did not reach statistical significance. In contrast, analyses of ORs based on proportions of anxiety symptoms did not demonstrate a significant association. One study that could not be included in the meta-analyses reported an elevated risk ratio for anxiety in dementia conversion [7]. No studies published from 2000 to 2006 were eligible for inclusion, which may reflect the increasing research interest in the association between anxiety and dementia.

To our knowledge, only one previous meta-analysis has examined dementia risk in relation to anxiety symptoms specifically among persons with MCI [13]. That systematic review reported significant, but lower HRs for dementia conversion in comparison to the present analysis and did not include studies published after 2015, nor did it report pooled ORs or mean differences in anxiety symptom severity. The present systematic review includes seven primary studies published after 2015. Other meta-analyses examining anxiety symptoms in cognitively unimpaired populations have similarly reported an increased risk of dementia conversion [33, 34], supporting the overall direction of the present findings, although pooled risk estimates vary across studies. The discrepancy between statistically significant HRs and nonsignificant ORs likely reflects methodological differences between time-to-event analyses and analyses based on conversion status. If anxiety is associated with earlier or faster conversion to dementia, this association would be easier to detect in time-to-event analyses.

The findings of the present meta-analysis are primarily generalizable to memory clinic populations, as most included studies were conducted in this setting. The prevalence of anxiety symptoms at baseline varied substantially across studies, although most reported relatively high proportions of anxiety. Differences in prevalence may in part be explained by heterogeneity in anxiety assessment tools and diagnostic thresholds. While the NPI-Q was mostly used, studies reporting the highest prevalence of anxiety used alternative instruments [6, 7, 23] whereas one study with particularly low prevalence used ICD-10 diagnostic criteria for anxiety disorders [29]. Nevertheless, the overall high prevalence of anxiety symptoms across studies supports the view that anxiety is among the most frequent NPS in persons with cognitive impairment.

Rates of progression from MCI to dementia were generally consistent with those reported in the literature, although some studies reported higher conversion rates [7, 15, 23, 27] while others reported comparatively low rates [24, 30, 31]. These differences could be due to variation in diagnostic criteria for MCI and dementia, differences in study populations, or duration of follow-up. Conversion rates in clinical settings are commonly reported to be approximately 10% per year [35], and this should be considered since most included studies had follow-up times of 3 years or less. Indeed, in one study with longer follow-up, ORs for dementia conversion varied substantially across different follow-up intervals, although none reached statistical significance [6].

Although pooled HRs indicated a statistically significant association between anxiety symptoms and conversion from MCI to dementia, most individual studies did not demonstrate statistically significant associations, with only a few exceptions [27, 28]. However, most studies reported point estimates suggestive of increased risk. The lowest HR and OR were reported by Peters et al. [25], which may be explained by an exceptionally low prevalence of anxiety symptoms in that cohort, resulting in limited statistical power and wide confidence intervals. Similarly, in the study by Yang et al. [30], anxiety symptoms were more frequent among non-converters than converters, although both anxiety prevalence and conversion rates were low, and the difference did not reach statistical significance.

Discrepancies between study findings may reflect differences in study design, population characteristics, sample size, diagnostic criteria for anxiety, MCI, and dementia, as well as methodological issues such as loss to follow-up in some cohorts [21, 27]. Another important consideration is that numerous of the included studies relied on anxiety symptoms assessed at a single time point (baseline) for risk estimation, which may lead to exposure misclassification and likely bias effect estimates toward the null. Supporting this, a recently published study by Khaing et al. [36] in community-dwelling adults aged 55–85 years, demonstrated that the association between anxiety and dementia was markedly attenuated when anxiety was assessed only at baseline compared with analyses using repeated measures over time.

The same study by Khaing et al. [36] also suggested that the association between anxiety and dementia progression may be modified by age, with significant associations observed only in persons aged 70 years or younger. This is consistent with previous findings indicating that anxiety in midlife increases the risk of dementia later in life [37]. Although evidence remains insufficient to draw firm conclusions regarding age-related effect modification, several of the studies included in this meta-analysis with younger mean participant ages [6, 24, 28] reported stronger associations than studies with older cohorts [25, 26].

The biological mechanisms linking anxiety symptoms to dementia progression remain incompletely understood. The most obvious explanation for the observed association is that anxiety symptoms simply reflect awareness of disease among persons diagnosed with a cognitive disorder. Another possibility is that anxiety represents an early neuropsychiatric manifestation of neurodegenerative pathology [38]. Anxiety could therefore reflect more advanced cognitive impairment given that the prevalence of NPS increases along the cognitive continuum [39]. However, chronic anxiety has been hypothesized to promote neurodegeneration through hypothalamic-pituitary-adrenal axis dysregulation [38], and anxiety in cognitive impairment has also been associated with increased axon/synaptic degeneration [40], biomarkers of AD in cerebrospinal fluid [41], moderation of cognitive decline in relation to brain amyloid-burden [42], and medial temporal lobe atrophy [11]. In addition, anxiety has been increasingly linked to cardiovascular disease [43], which in turn is a risk factor for cognitive impairment and decline [44]. A recent study found an association between white matter hyperintensity volume and anxiety, although it remains unclear whether anxiety contributes causally to cerebral small-vessel disease and other cerebrovascular pathologies, rather than reflecting reverse causation [45]. The study by Khaing et al. [36] addressed the concerns regarding reverse causality through a sensitivity analysis excluding early cases of dementia and death, with largely unchanged effect estimates. Moreover, evidence of a dose-response relationship between anxiety severity and dementia risk could possibly support anxiety as a causal risk factor, although confidence intervals in these analyses were wide. The potential confounding effect of depressive symptoms should also be considered, given the overlap between anxiety and depression. However, findings across studies have been inconsistent, with some reporting associations for depression and dementia conversion but not anxiety [15, 26] and others reporting the opposite [28]. A study of persons with amnestic MCI found that anxiety was associated with medial temporal lobe atrophy and incident dementia, when controlling for depression [11]. Therefore, it is unlikely that confounding by depression alone can explain the observed associations. Finally, in a longitudinal study of over 1,000 twins, anxiety symptoms were associated with incident dementia, although shared genetic factors partially explained this effect [46].

Although a statistically significant association between anxiety and dementia progression was observed, this does not necessarily translate into a clinically meaningful predictive marker. The observed standardized mean difference of 0.16 indicates a small difference in anxiety symptom severity between converters and non-converters [47]. Moreover, anxiety is a nonspecific symptom that occurs in a wide range of medical and psychiatric conditions, including major depressive disorder [48], heart failure [49], stroke [50], multiple sclerosis [51], and substance-related disorders such as alcohol withdrawal [52]. The clinical utility of anxiety as a predictor of dementia progression is therefore uncertain, particularly given that commonly used instruments for assessing NPS, including the NPI-Q, have not been formally validated specifically in MCI populations.

Multiple pharmacological and non-pharmacological treatment options are available for anxiety, including antidepressant medications, anxiolytics, psychotherapy, and lifestyle interventions such as physical activity. In a population of memory clinic patients, memantine has shown only modest effects in reducing NPSs and does not appear to improve anxiety [53]. Moreover, the effect of treating anxiety on dementia risk remains unclear, and a cohort study based on the Swedish dementia register instead showed increased rates of dementia in persons treated with selective-serotonin reuptake inhibitors [54].

Limitations of this systematic review and meta-analysis include: (1) HRs could not be obtained from all included studies, limiting inclusion in the primary analysis, (2) although adjusted HRs were used when available, the variables included in adjustment models varied across studies, (3) reliance on baseline assessments of anxiety may have resulted in exposure misclassification, (4) uncertain completeness of follow-up in some cohorts, i.e., attrition bias, and (5) few studies could be included in some of the subgroup analyses, which reduces the certainty of these estimates, (6) the robustness of the primary analysis was affected since the result was no longer statistically significant after leaving one out analysis, (7) anxiety was defined using varying scales and criteria and the pooled estimates from the analyses are therefore based on heterogenous definitions of anxiety; this is why the mean difference in symptom scores is expressed as standardized mean difference. Some of the strengths of this systematic review and meta-analysis are that it follows the PRISMA guidelines, the literature search was conducted by a trained librarian, and predefined inclusion and exclusion criteria were used. Another strength of meta-analyses, including the present one, is that data from small or underpowered studies can be pooled to increase statistical power, allowing the detection of associations that may not be apparent in individual studies.

In conclusion, the findings of this systematic review and meta-analysis suggest that anxiety symptoms may be associated with an increased risk of progression from MCI to dementia, although effect sizes are modest, the leaving one out analysis for the primary outcome was no longer statistically significant, and clinical significance remains uncertain. The certainty of evidence was rated as very low, indicating that it is uncertain whether the pooled estimates reflect the true effect, and that future research is likely to change these estimates. Further large prospective cohort studies using repeated assessments of anxiety are needed to clarify the role of anxiety and other NPS in predicting progression from MCI to dementia and to determine whether these could represent modifiable risk factors.

## Acknowledgments

The authors would like to thank information specialist Marika Svalstedt at the Hospital Library, Central Hospital Karlstad, for excellent assistance in performing the literature search for this systematic review and meta-analysis. ChatGPT (OpenAI, GPT-5) was used solely for language refinement of the manuscript. It was not involved in the study design, search strategy, study selection process, data extraction, data analysis, assessment of quality of individual studies, generation of ideas or content, interpretation of results, or scientific conclusions – these were all performed by the authors.

## Statement of Ethics

This systematic review and meta-analysis followed Swedish law and guidelines of the Ethical Committee of Uppsala University, Sweden. A patient consent statement is not applicable to this systematic review and meta-analysis as it is based entirely on previously published literature.

## Conflict of Interest Statement

We have no conflicts of interest to disclose.

## Funding Sources

This study was conducted as part of the first author’s PhD studies. The first author’s salary is funded by (1) Örebro University, School of Medical Sciences, Örebro, Sweden, and (2) Region Värmland, through employment at Karlstad Central Hospital, Karlstad, Sweden. The funders had no role in the study conception, design, execution, analysis, writing or decision to publish – all aspects of the work were performed independently by the authors.

## Author Contributions

This study was designed and conceived by David Fresnais and Brynjar Fure. Both investigators helped with data extraction, assessment of quality of included studies and in performing meta-analyses. The first draft of the manuscript was written by David Fresnais and later reviewed by Brynjar Fure. Finally, a revised version of the manuscript was written by David Fresnais.

## Data Availability

This study is based exclusively on data from previously published works, which are cited in this article. All data generated or analyzed during this systematic review and meta-analysis are included in this article, including its online supplementary material files. Further inquiries can be directed to the corresponding author. See the online Supporting Information for additional details regarding the article search strategy and selection process, as well as the PRISMA Checklist.
